# Expression of *PAX8* Target Genes in Papillary Thyroid Carcinoma

**DOI:** 10.1371/journal.pone.0156658

**Published:** 2016-06-01

**Authors:** Francesca Rosignolo, Marialuisa Sponziello, Cosimo Durante, Cinzia Puppin, Catia Mio, Federica Baldan, Carla Di Loreto, Diego Russo, Sebastiano Filetti, Giuseppe Damante

**Affiliations:** 1 Department of Internal Medicine and Medical Specialties, “Sapienza” University of Rome, 00161 Rome, Italy; 2 Department of Medical and Biological Sciences, University of Udine, 33100 Udine, Italy; 3 Department of Oncology, University Hospital “S. Maria della Misericordia”, 33100 Udine, Italy; 4 Department of Health Sciences, University “Magna Graecia” of Catanzaro, 88100 Catanzaro, Italy; University Claude Bernard Lyon 1, FRANCE

## Abstract

PAX8 is a thyroid-specific transcription factor whose expression is dysregulated in thyroid cancer. A recent study using a conditional knock-out mouse model identified 58 putative *PAX8* target genes. In the present study, we evaluated the expression of 11 of these genes in normal and tumoral thyroid tissues from patients with papillary thyroid cancer (PTC). *ATP1B1*, *GPC3*, *KCNIP3*, and *PRLR* transcript levels in tumor tissues were significantly lower in PTCs than in NT, whereas *LCN2*, *LGALS1* and *SCD1* expression was upregulated in PTC compared with NT. Principal component analysis of the expression of the most markedly dysregulated *PAX8* target genes was able to discriminate between PTC and NT. Immunohistochemistry was used to assess levels of proteins encoded by the two most dyregulated *PAX8* target genes, LCN2 and GPC3. Interestingly, GPC3 was detectable in all of the NT samples but none of the PTC samples. Collectively, these findings point to significant PTC-associated dysregulation of several *PAX8* target genes, supporting the notion that PAX8-regulated molecular cascades play important roles during thyroid tumorigenesis.

## Introduction

Tissue-specific transcription factors are critical for the development and function of the thyroid gland. Several thyroid-specific transcription factors have been identified, including TTF-1 (NKX2-1), TTF-2 (FOXE1), PAX8, and HEX, and numerous roles have been described for each [1]. PAX8 is a member of the PAX protein family [2] and interacts with specific DNA sequences via its paired domain [3]. Its critical contribution during thyroid development was first highlighted by Mansouri and coworkers, who demonstrated the absence of thyroid follicular cell formation in *Pax8* knock-out mice [4]. Consistently, most cases of human congenital hypothyroidism due to thyroid dysgenesis are caused by heterozygous loss-of-function mutations involving *PAX8* [5]. PAX8 also appears to control the expression of various genes that play key roles in the function of thyroid follicular cells, including those encoding thyroglobulin (*TG*), thyroperoxidase (*TPO*), and the sodium-iodide symporter (*NIS*, also known as solute carrier family 5, member 5, *SLC5A5*) [1,6,7]. A recent report by Marotta et al. confirms that *Pax8* is also essential for post-natal thyroid function. Mice subjected to conditional *Pax8* knock-out exhibited undetectable serum levels of T4 and significantly increased levels of TSH. Moreover, the thyroid glands of these animals were characterized by the absence of follicular structure and dedifferentiation of the follicular cells, and they were significantly smaller than those of control animals. The authors identified a set of 58 genes whose expression was dysregulated after *Pax8* knock-out and suggested that they might be used to delineate the molecular cascades underlying PAX8’s regulation of thyroid follicular cell function [8].

Extensive work has been done to characterize *PAX8*’s role in thyroid carcinomas [6,9–12]. The general notion emerging from these studies is that PAX8 expression is frequently downregulated in thyroid carcinoma and that this decrease may correlate with the dedifferentiation of thyroid follicular cells, reflected by the loss or downregulated expression of genes involved in the cells’ ability to concentrate iodine [10], thereby contributing to tumor aggressiveness [11]. In addition, the *PAX8–PPARγ* fusion gene has been found in roughly one-third of all follicular thyroid carcinomas and a small fraction of follicular-variant papillary thyroid carcinomas (PTCs) as well, but it is not present in classical PTCs [12].

To gain further insight into the roles played by *PAX8* target genes during thyroid tumorigenesis, we investigated their expression in a cohort of PTCs with well characterized clinicobiological features.

## Materials and Methods

The study was conducted with the approval of the Bioethics Committees of both participating centers (Sapienza University of Rome, Policlinico Umberto I and the University of Udine, Santa Maria della Misericordia Hospital). All tissue donors provided written informed consent to the collection and analysis of tissue samples and clinical data and to the publication of the results of the study. Unless otherwise stated, all commercial products mentioned below were used in accordance with the manufacturers’ instructions.

### Patient and samples

*PAX8* mRNA levels were assessed in surgical specimens of 36 PTCs collected between 2008 and 2014 at the University of Rome. All have been analyzed in previous reports [13,14]. Thirty-one of the tumors were classical-type (CT-PTCs) and the remaining five were follicular-variants (FV-PTCs). Specimens of normal thyroid tissue from the tumor-free lobe were also tested for 18 of the 36 PTCs (15 CT-PTCs, 3 FV-PTCs). All tissues were immediately snap-frozen and stored in liquid nitrogen prior to use. A single experienced pathologist reviewed all tissues to confirm the diagnosis of PTC and select samples suitable for use in the study (i.e., tumor tissue samples with a percentage of tumor cells exceeding 60%, normal tissues exhibiting no signs of hyperplasia or thyroiditis). Each case was staged using the AJCC/UICC TNM classification [15] and risk-stratified on the basis of the clinical and histological criteria recommended by current American Thyroid Association (ATA) guidelines [16].

For immunohistochemistry studies, we used an archival series of 38 PTCs (all CT-PTCs) and 12 NTs from the the University of Udine. The most representative block of each lesion was retrieved from the archive and used for our analyses.

### Evaluation of mRNA levels for thyroid-specific genes and PAX8 target genes

Total RNA was isolated from tissue samples using Trizol reagent (Thermo Fisher Scientific, Waltham, MA, USA), and first-strand cDNA was synthesized with the High Capacity cDNA Reverse Transcription kit (Thermo Fisher Scientific). Gene expression profiling of thyroid tissues was done by real-time PCR with custom Taqman Low Density Arrays (TLDA, Thermo Fisher Scientific), each configured with predesigned assays (TaqMan Gene Expression Assays, Life Technologies) for six thyroid-specific genes (*SLC5A5*, *TPO*, *TG*, *TSHR*, *TTF1*, *PAX8*) and 11 of the 58 putative PAX8-target genes identified by Marotta et al. on the basis of their studies in *PAX-8* knock-out mice, namely: *FSTL1*, *LCN2*, *CA3*, *KCNIP3*, *PRLR*, *NFKBIA*, *GPC3*, *LUM*, *LGALS1*, *SCD1*, *ATP1B1*. The latter 11 genes were selected on the basis of the degree of dyregulation they displayed in the mouse studies and their established roles in thyroid cell function or in thyroid cancerogenesis [8]. Four housekeeping genes (glyceraldehyde-3-phosphate dehydrogenase; beta-actin; hypoxanthine phosphoribosyltransferase 1, and beta-2 microglobulin) were included in each reaction and tested as endogenous controls. The TaqMan arrays were processed and analyzed on a 7900HT Fast Real-Time PCR System (Thermo Fisher Scientific), as previously described [17]. The relative expression of each transcript was determined by the comparative 2-ΔΔCt method using RQ Manager 1.2.1 software (Thermo Fisher Scientific). Beta-actin was chosen as the endogenous control because of the stability of its expression levels among samples. Final results were expressed as means ± standard deviation.

### Detection of the *BRAF*^*V600E*^ mutation

We analyzed cDNA from tumor tissues for the presence of the *BRAF*^*V600E*^ mutation. The PCR reaction was performed on 100 ng of cDNA using 200 mM dNTPs, 10 pmol of specific primers for exon 15 of *BRAF* (Fw: 5ʹ-CACAGAGACCTCAAGAGTAA-3ʹ, Rv: 5ʹ-ATGACTTCTGGTGCCATCCA-3ʹ), 1.5 mM MgCl2, 1 U of AmpliTaq Gold, and Buffer 1x AmpliTaq Gold DNA Polymerase (Thermo Fisher Scientific). The cycling conditions for PCR program included 10 min at 95°C, followed by 35 cycles (each consisting of 30 sec at 95°C, 30 sec at 58°C, and 30 sec at 72°C), and a final 7 min extension at 72°C.

All PCR products were purified with the NucleoSpin Gel and PCR Clean-up Kit (Macherey-Nagel GmbH & Co. KG, Düren, Germany), and sequenced using one of the primers described above and the Big DyeTM Terminator v. 3.1 Cycle Sequencing Kit (Thermo Fisher Scientific). The products of the sequencing reaction were purified with NucleoSeq Columns (Macherey-Nagel GmbH & Co. KG) and analyzed with an Applied Biosystems 3130 XL Automated Sequencer (Thermo Fisher Scientific). This protocol has been shown to detect mutations with allelic frequencies as low as 10% [18].

To confirm the presence of a *BRAF*^*V600E*^ mutation, we repeated PCR and sequencing reactions at least twice.

### Collection of TCGA public data

Gene expression data on 486 PTCs and 59 normal thyroid tissues were downloaded from http://gdac.broadinstitute.org/(gdac.broadinstitute.org_THCA.Merge_rnaseqv2__illuminahiseq_rnaseqv2__unc_edu__Level_3__RSEM_genes_normalized__data.Level_3.2015060100.0.0.tar). Matching clinical data were downloaded from http://www.cbioportal.org/ (Papillary Thyroid Carcinoma (TCGA, Cell 2014).csv). RNA-Seq data were expressed as RNA-seq by Expectation Maximization (RSEM) values [19].

### Immunohistochemistry

Antibodies against PAX8, LCN2, and GPC3 were purchased from Abcam (Cambridge, UK), Sigma Aldrich (St. Louis, MO, USA) and Aczonpharma (Bologna, Italy), respectively. Formalin-fixed paraffin-embedded tissue sections (5 μm) mounted on SuperFrost Plus slides (Menzel-Gläser, Braunschweig, Germany) were placed in the PT Link Pre-Treatment module (DAKO A/S, Glostrup, Denmark), which automatically performs the entire pre-treatment process, including deparaffinization, rehydration, and epitope retrieval, the latter done with DAKO’s Low pH Target Retrieval Solution (0.001 M citrate buffer pH 6.0) at 98°C for 40 min. Endogenous peroxidase activity was blocked by a 5-min incubation in the Peroxidase Block solution (DAKO). Primary antibodies (dilutions: 1:30 for PAX8, 1:500 for LCN2, 1:50 for GPC3) were applied and sections incubated for 60 minutes at room temperature. After washing, slides were incubated in the DAKO EnVision FLEX System (DAKO) and the reaction visualized using 3–3 diaminobenzidine tetrahydrochloride as the chromogen. The sections were counterstained with Mayer hematoxylin. Appropriate positive and negative tissue controls were run for each antibody. Brown-colored cells were identified as positive.

PAX8 staining intensity was semiquantitatively rated using the H-score method [20]. The H score for each sample was determined independently by two experienced pathologists, and the average used for all analyses.

### Statistical Analysis

Analysis of differential expression between groups was based on the Mann–Whitney test. When more than two groups were compared, we used the Kruskal–Wallis test with the post hoc Dunn’s multiple comparison test. Correlation analysis was performed with the Spearman’s rho rank correlation coefficient. GraphPad Prism version 5.0 statistical software was employed for all the above analyses. P-values < 0.05 were considered statistically significant. Bonferroni correction for multiple comparisons was used to evaluate adjusted p values (adj p-value). Principal component analysis (PCA) was carried out with the prcomp function from the built-in stats package in R software v.3.1.1, which uses the singular value decomposition (SVD). Receiver-operating characteristic (ROC) curves and areas under the ROC curve (AUC) were analyzed with the pROC package on R software, v.3.1.1, as previously reported [21,22], using the Youden Index and the DeLong method.

## Results

### PAX8 expression in Normal Thyroid and PTC

**Fig 1:**
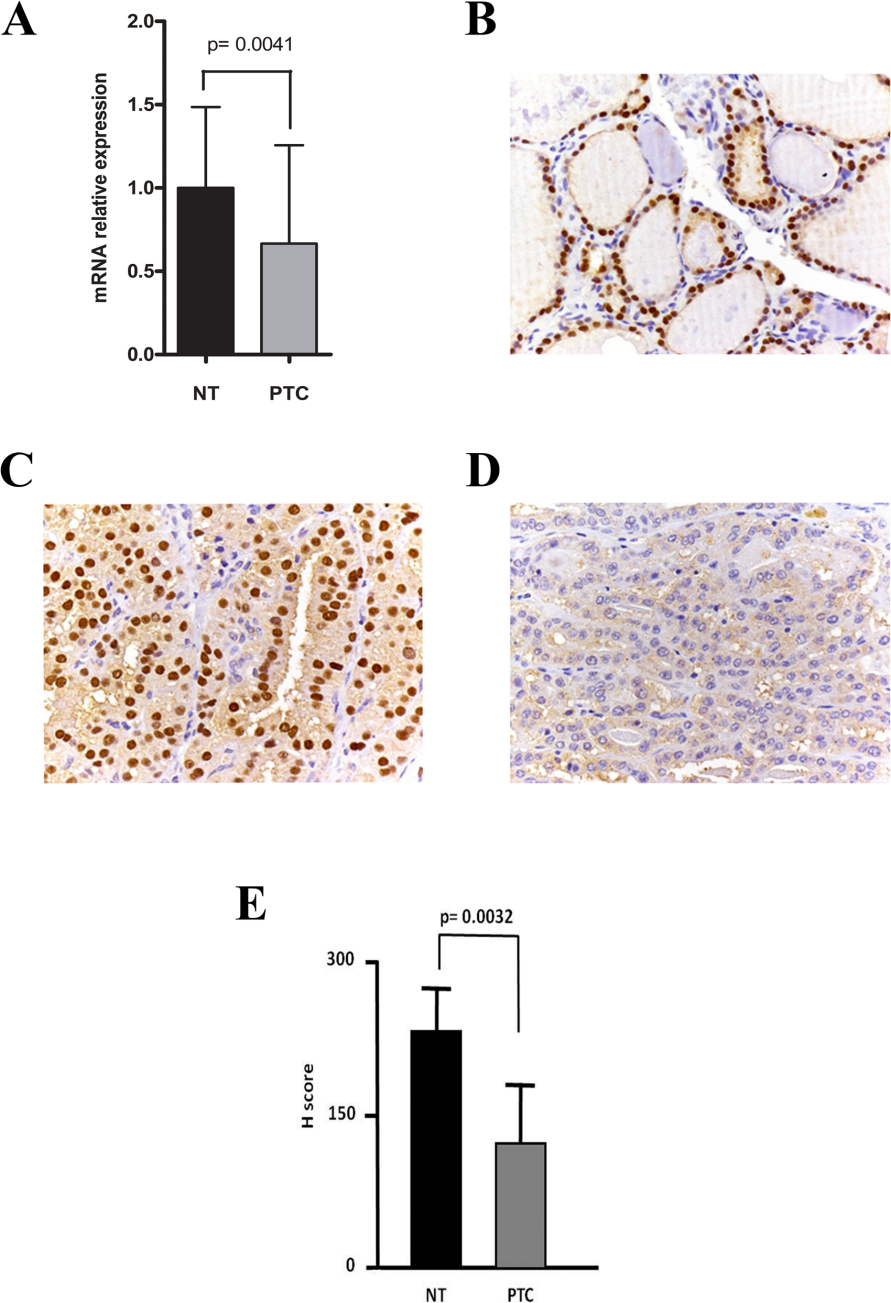
*PAX8* expression in normal and tumor thyroid tissues of PTC patients. (A) Mean (SD) *PAX8* mRNA levels found in normal thyroid tissuesamples (n = 18) and PTCs (n = 36), assessed by qPCR. (B-D) Representative images of immunohistochemical staining for PAX8 protein levels in (B) normal thyroid tissues; (C) PAX8-positive PTCs; and (D) PAX8-negative PTCs. (E) Mean H scores for immunohistochemical labeling of PAX8 protein in normal thyroid tissues (12 samples) and PTC (38 samples).

Analysis of mean *PAX8* mRNA levels in the 36 PTCs and 18 normal thyroid tissue (NT) samples revealed significantly reduced expression in the tumors (Fig 1A). A similar picture emerged when PAX8 protein levels in the second (archival) cohort of PTC and NT samples were assessed by immunohistochemistry: all 12 NT samples showed strong PAX8 positivity, whereas PTCs were either moderately (21 samples) or weakly (17 samples) positive (Fig 1B and 1D). The mean H score for the NT group was significantly higher than that of the PTCs (Fig 1E).

*PAX8* gene expression in the PTCs displayed strong correlation with *TG*, *TSHR*, and *TTF1* expression and weak correlation with expression of the iodine transporter gene *SLC5A5*. No correlation was observed between *PAX8* and *TPO* mRNA levels. (S1 Fig).

### mRNA levels of PAX8 target genes in NT and PTC

As shown in Table 1, the 11 putative *PAX8*-target genes investigated in the present study included four (*ATP1B1*, *GPC3*, *KCNIP3*, and *PRLR*) with significantly lower mRNA levels in PTCs than in NTs and three (*LCN2*, *LGALS1*, and *SCD1*) that displayed significantly upregulated expression in the tumors, as compared with NT. The dysregulation of *GPC3*, *KCNIP3*, *LCN2*, *LGALS1*, and *SCD1* remained significant after correction for multiple testing (Bonferroni-adjusted *P* values < 0.05). These five genes displayed directionally similar dysregulations in PTCs from The Cancer Genome Atlas (TCGA) dataset, which were highly significant (p values ranging from 1.82x10^-12^ for *SCD1* to 6.04x10^-22^ for *LCN2*) (data not shown).

**Table 1:** Expression of putative PAX8 target genes in the 36 PTCs.

Gene	NT(n = 18)	PTC(n = 36)	p-value	adjp-value
Upregulated genes in Pax8 knock-out mice*				
***CA3***	1±0.462	1.273±0.721	ns	ns
***FSTL1***	1±0.496	1.068±0.861	ns	ns
***GPC3***	1±0.626	0.067±0.091	<0.0001	0.0011
***LCN2***	1±0.857	44.400±78.410	<0.0001	0.0011
***LGALS1***	1±0.643	2.793±2.941	0.0023	0.0253
***LUM***	1±0.743	2.596±8.738	ns	ns
***SCD1***	1±0.948	3.158±2.682	<0.0001	0.0011
Downregulated genes in Pax8 knock-out mice*				
***ATP1B1***	1±0.493	0.720±0.330	0.0318	ns
***KCNIP3***	1±0.829	0.355±0.368	<0.0001	0.0011
***NFKBIA***	1±0.429	1.100±1.268	ns	ns
***PRLR***	1±0.820	0.411±0.302	0.0017	ns

mRNA levels are expressed as mean ± SD.

p values were obtained by Mann-Whitney test.

adj p-value: adjusted p-values were evaluated using the Bonferroni method.

NT, Normal Thyroid; PTC, Papillary Thyroid Carcinoma.

* Data from Marotta et al., 2014.

Notably, *FSTL1*, *LCN2*, *LGALS1*, and *SCD1* showed significantly higher mRNA levels in CT-PTC than in FV-PTC. Compared with NTs, FV-PTCs displayed downregulated expression of *FSTL1* and *GPC3*, and CT-PTCs exhibited significant dysregulation (up or down) of *GPC3*, *KCNIP3*, *LCN2*, *LGALS1*, *PRLR*, and *SCD1* (S1 Table).

**Fig 2:**
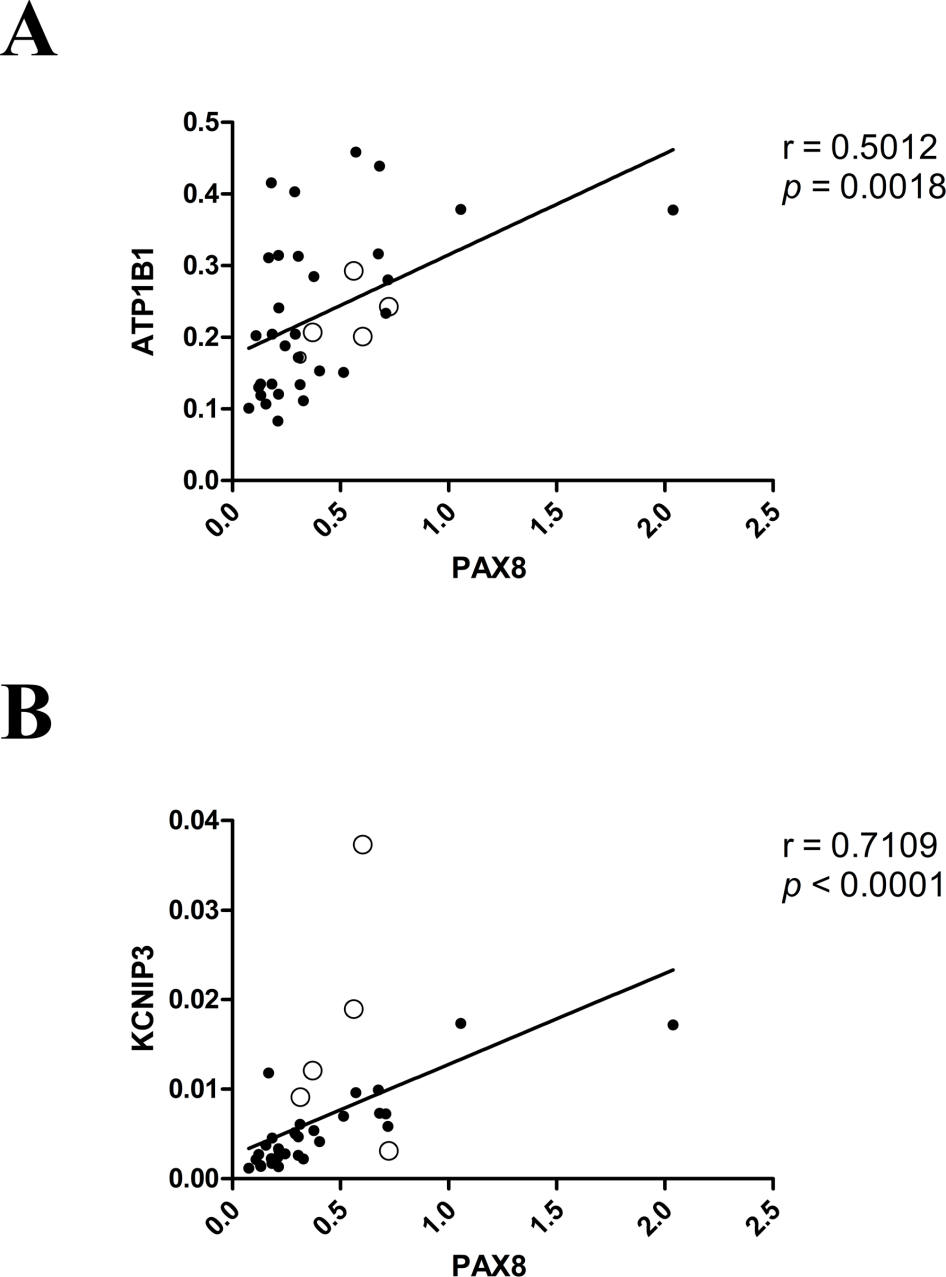
**Putative PAX8 target genes whose mRNA levels were most strongly correlated with those of *PAX8* in PTCs** Levels of mRNA for *ATP1B1* (A) and *KCNIP3* (B) are plotted against PAX8 mRNA levels measured in 31 CT-PTCs (•) and 5 FV-PTCs (○). R and p values were calculated with the Spearman rank correlation test.

Interestingly, for six of the 11 putative *PAX8* target genes, the dysregulation we observed in PTCs (vs. NT) was directionally consistent with that reported in the thyroids of *PAX8* knock-out mice (Table 1). These included three of the genes displaying PTC-related downregulation (*ATP1B1*, *KCNIP3*, and *PRLR*) and three of those that were upregulated in the tumors (*LCN2*, *LGALS1*, *SCD1*). The genes whose expression showed the strongest correlation with *PAX8* mRNA levels in the PTC group were *ATP1B1* and *KCNIP3* (Fig 2).

*NFKBIA* expression was also strongly correlated with *PAX8* mRNA levels in the PTCs (S2 Table). However, mRNA levels for this gene in NTs and PTCs were not significantly different (Table 1). As for *GPC3*, its downregulation in PTCs vs. NT (Table 1) contrasts with the upregulation reported in PAX-8 knock-out mice. Collectively, these data indicate that in PTCs, PAX8-dependent regulation is conserved for only some of the target genes of this transcription factor.

None of the 11 genes exhibited significantly different expression levels in PTCs with (n = 21) vs. without (n = 15) the *BRAF*^*V600E*^ mutation or in PTCs belonging to low (n = 17) vs. intermediate (n = 19) risk groups (Table 2).

**Table 2:** Expression of putative PAX8 target genes in the 36 PTCs stratified by ATA risk and *BRAF* mutational status.

Gene	*ATA risk		**BRAF status	
	Low Risk (n = 17)	Intermediate Risk (n = 19)	*BRAF–wt* (n = 21)	*BRAF*^*V600E*^ (n = 15)
**Upregulated genes in Pax8 knock-out mice**^§^				
***CA3***	1±0.556	0.930±0.550	1±0.535	0.956±0.577
***FSTL1***	1±0.824	1.376±1.067	1±0.857	0.926±0.724
***GPC3***	1±1.236	0.719±1.092	1±1.178	0.653±1.011
***LCN2***	1±1.234	1.637±3.033	1±1.103	1.985±3.495
***LGALS1***	1±1.329	1.293±1.112	1±1.172	0.903±0.858
***LUM***	1±3.374	0.417±0.531	1±1.635	2.524±8.240
***SCD1***	1±0.904	0.772±0.573	1±1.057	1.707±1.246
**Downregulated genes in Pax8 knock-out mice**^§^					
***ATP1B1***	1±0.447	0.870±0.412	1±0.440	1.065±0.509
***KCNIP3***	1±0.649	0.944±1.265	1±1.000	0.531±0.448
***NFKBIA***	1±1.307	0.805±0.736	1±1.125	0.580±0.602
***PRLR***	1±2.047	0.571±0.771	1±1.559	0.178±0.126

mRNA levels are expressed as mean ± SD.

* mRNA levels in the ATA risk intermediate group are expressed as relative quantity with respect to the ATA risk low group, arbitrarily considered as 1.0.

** mRNA levels in samples with the *BRAF*^*V600E*^ mutation are expressed as relative quantity with respect to *BRAF-wt* group, arbitrarily considered as 1.0.

All mRNA values indicated in this table have p values >0.05 when compared to respective control groups, as assessed by Mann Whitney test.

wt, wild type.

^§^ Data from Marotta et al., 2014.

To determine whether the lack of significant differences in the last two analyses was due to the low number of PTCs in each subgroup (i.e. *BRAF-wt*, *BRAF*^*V600E*^, low risk, and intermediate risk tumors), we assessed mRNA expression levels for the 11 *PAX8* target genes present in the TCGA dataset. This analysis showed that, compared with *BRAF-wt* PTCs, those harboring the *BRAF*^*V600E*^ mutation displayed significantly higher expression of *FSTL1*, *LCN2*, *LGALS1*, *LUM*, and *SCD1* and significantly lower expression of *GPC3*, *KCNIP3*, and *NFKBIA*. With the exception of *GPC3*, all of these genes continued to display significant differential expression in *BRAF-wt* and *BRAF*^*V600E*^ PTCs after Bonferroni correction (S3 Table). Analysis of the TCGA dataset also revealed significant differential expression in PTCs of *FSTL1*, *KCNIP3*, *LCN2*, *LGALS1*, *LUM*, and *SCD1* related to ATA risk statuses. After correction for multiple testing, the most striking differences were observed for the *LCN2* gene, which displayed higher expression in intermediate- and high-risk PTCs compared with those considered low-risk (fold change 2.384, adjusted p-value<0.001, and fold change = 2.568, adjusted p-value<0.001, respectively) (S2A Fig, S4 Table). These findings prompted us to subject *LCN2* to ROC analysis to assess its accuracy in discriminating high risk-PTC from low risk-PTC. The diagnostic accuracy of this potential marker was mediocre, however, with an AUC of 77.3% [95% CI of 68.64–85.88%], specificity of 62%, sensitivity of 83.3%, and accuracy of 64.7% (S2B Fig).

**Fig 3:**
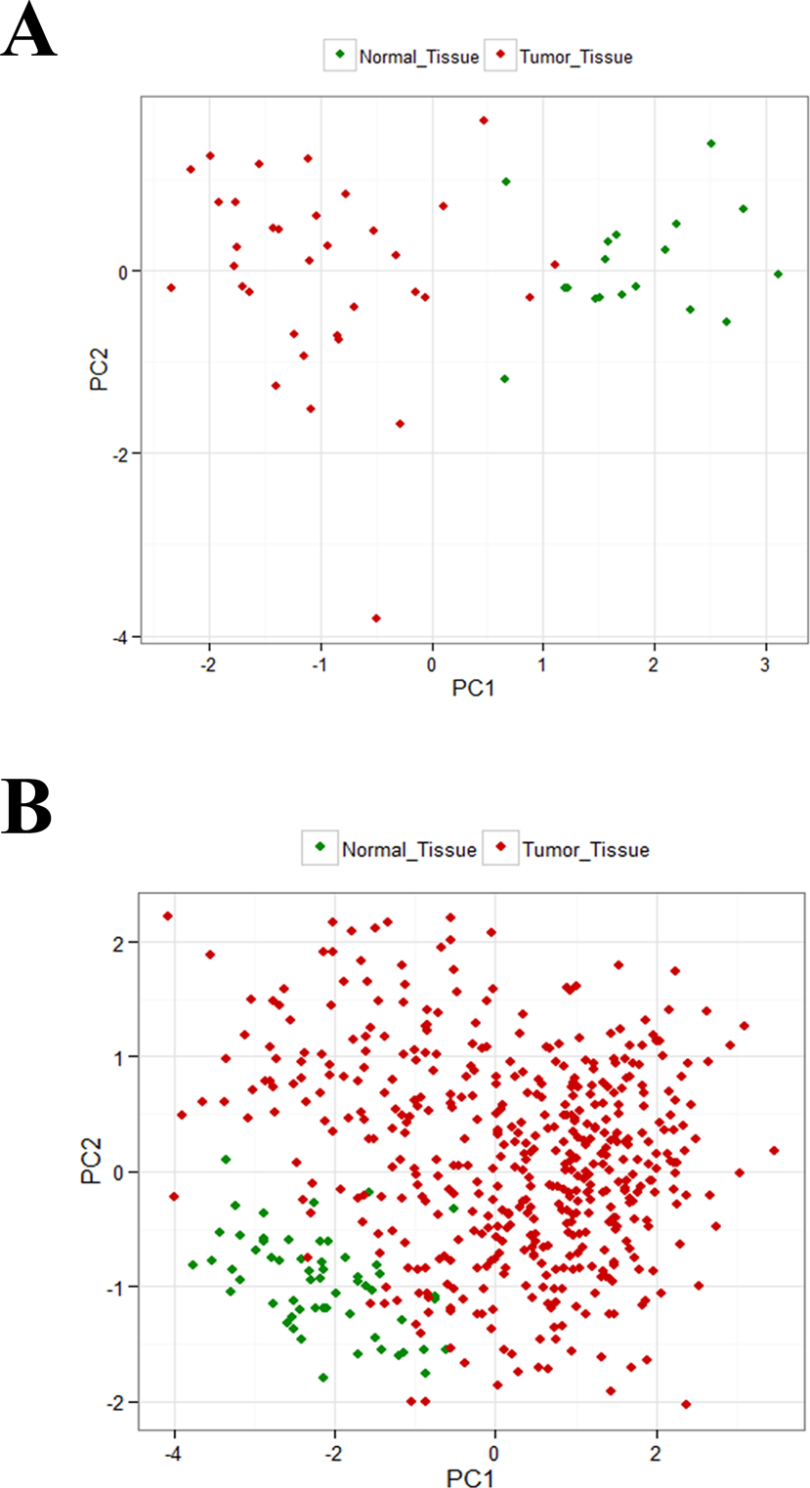
PCA of PAX8 target genes displaying marked PTC-related dysregulation. Analysis was performed with data for the four *PAX8* target genes (*LCN2*, *GPC3*, *KCNIP3*, *SCD1*) that were most markedly dysregulated in PTCs (red diamonds) compared with normal thyroid (NT) tissues (green diamonds). (A) Our dataset (36 PTC and 18 NTs). (B) TCGA dataset (486 PTC and 59 NTs). PCA was performed with the built-in prcomp function in R software.

To determine whether dysregulation of PAX8 target genes can discriminate between NT and PTCs, expression levels of the four most markedly dysregulated genes (*LCN2*, *GPC3*, *KCNIP3* and *SCD1*) in our dataset were subjected to principal component analysis (PCA). As shown in Fig 3A, NT and PTC clearly occupy different positions within the PCA space. To validate this finding, we repeated the PCA using expression levels of *LCN2*, *GPC3*, *KCNIP3* and *SCD1* found in the TCGA dataset. Again, the positions of NT and PTC were distinct (Fig 3B). Expression levels of the PAX8 target genes most markedly dysregulated in PTCs effectively discriminates between these tumors and NT, suggesting similarity in dysregulatory mechanisms among PTC.

### Immunohistochemical detection of LCN2 and GPC3

**Fig 4:**
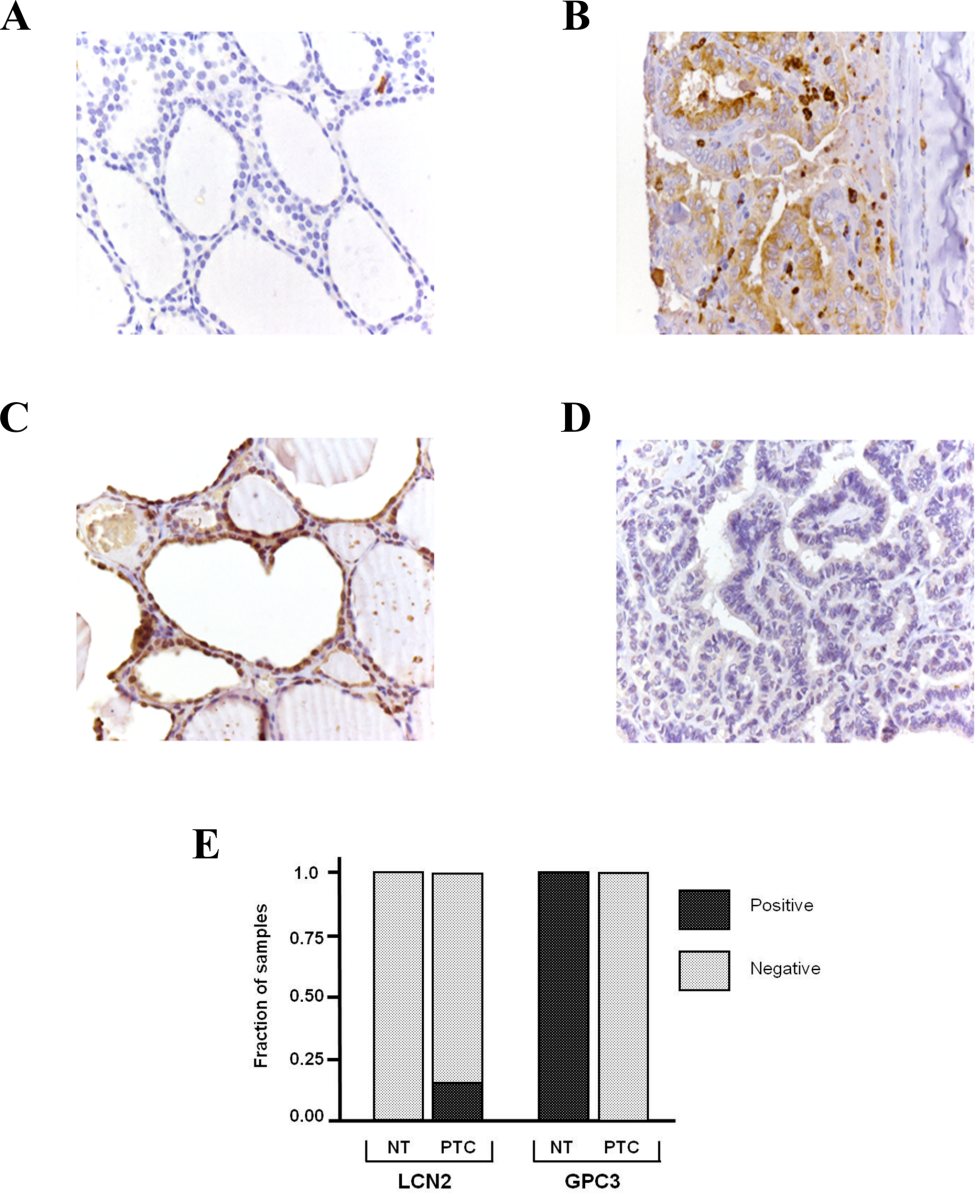
Immunohistochemical detection of LCN2 and GPC3. (A) LCN2 staining in normal thyroid. (B) LCN2 staining in PTC. (C) GPC3 staining in normal thyroid. (D) GPC3 staining in PTC. (E) Proportions of positive and negative samples in each tissue group.

At the transcriptional level, *LCN2* and *GPC3* were the PAX8 target genes with the most markedly altered expression in PTCs relative to NTs (upregulation expression for *LCN2*, downregulation for *GPC3*). We therefore focused our subsequent efforts on these two genes: immunohistochemistry was used to assess LCN2 and GPC3 protein levels in a different cohort of tissues, which included 12 NT samples and 38 PTCs. Representative results are shown in Fig 4. LCN2 was undetectable in all of the NTs and 31 of the 38 PTCs. These results were not completely surprising: in the Human Protein Atlas (http://www.proteinatlas.org/), negative immunohistochemical findings for LCN2 protein expression are reported in normal and cancerous thyroid tissues. LCN2 protein levels in most samples are probably below the detection limit of immunohistochemical techniques. Only in a few PTCs were mRNA levels increased sufficiently to produce protein levels detectable by antibody staining. As for GPC3 protein, it was detectable in all NTs but in none of the PTC samples (Fig 4). Thus, GPC3 staining is a good candidate for discriminating between NT and PTC.

## Discussion

Several findings indicate that expression of the thyroid-specific transcription factor PAX8 is reduced in thyroid cancer [23]. Thus, investigation of its targets could improve our understanding of the mechanisms of thyroid tumorigenesis. In addition to *TG*, *TPO*, and *SLC5A5* genes [1,7,24], a large number of other putative *Pax8* target genes have recentely been identified based in studies in mouse conditional knock-out model [8]. Here, we evaluated the expression in NT and PTC of 11 of these genes with established roles in thyroid cell function or in cancerogenesis, which displayed marked dysregulation in the Pax8 knock-out mice [8]. Five of the 11 genes (*GPC3*, *KCNIP3*, *LCN2*, *LGALS1*, and *SCD1*) presented significantly modified expression in PTCs relative to NT (Mann-Whitney test with Bonferroni adjustment), and all five displayed consistently dysregulated expression in the TCGA dataset, strenghtening the hypothesis that a substantial proportion of *PAX8* target genes are involved in PTC tumorigenesis. We then looked at the two genes displaying the most markedly upregulated and downregulated expression in the tumors (*LCN2* and *GPC3*, respectively). *LCN2* encodes lipocalin 2 or neutrophil gelatinase associated lipocalin (*NGAL*), a secreted 25-kDa protein that binds iron [25] and is overexpressed in various epithelial cancers [26]. Several findings indicate that *LCN2* overexpression is associated with tumor size, stage, and invasiveness [27], and it has been proposed as a negative prognostic indicator in several types of cancer [28,29]. We found that *LCN2* mRNA levels were markedly higher in PTCs than in NTs, and immunostaining for LCN2 protein was positive in several tumors but in none of the NT samples. Ma and coworkers recently showed that *LCN2* is overexpressed in PTC relative to NT [30]. Moreover, *LCN2* expression has been proposed as an efficient marker for differentiating benign from malignant thyroid neoplasms [31]. Consistent with these findings, in vitro experiments have shown that *LCN2* is a survival factor for thyroid neoplastic cells and that its overexpression increases the cells’ metastatic potential [32,33]. The mechanism by which *LCN2* contributes to thyroid tumorigenesis is not known, but studies involving other neoplasms suggest that it might be related to the protein’s promotion of epithelial-mesenchymal transition and/or its ability to sequester iron [25,26]. In our samples, however, *LCN2* mRNA levels were not significantly related to ATA risk groups. This finding may be due to the limited number of intermediate-risk cases and the absence of high-risk tumors in our cohort. Indeed, in the TCGA dataset, mean *LCN2* mRNA values are significantly different in low risk groups, relative to high-risk as well as to intermediate-risk groups (S2A Fig, S4 Table). In this analysis, only the mean *LCN2* transcript levels were correlated with aggressiveness: single *LCN2* mRNA levels were not an efficient indicator of ATA high risk (S2B Fig).

*GPC3* encodes the glypican-3 protein, which is a membrane-bound heparan sulfate proteoglycan. The biological role of GPC3 is linked to the Hedgehog (Hh) signaling pathway [34]. In fact, Hh signaling activity has been shown to be elevated in GPC3-null mice [35,36]. Moreover, GPC3 binds with high affinity to both Sonic Hedgehog and Indian Hedgehog and competes with Patched for Hh binding [35,36]. Conflicting data have been reported on the role of GPC3 in cancer. In fact, while this gene is overexpressed in liver cancer and germ-cell malignancies [37], other studies indicate that it is silenced in breast and lung cancers, where it is thought to play the role of a tumor suppressor [38,39]. The latter conclusion is consistent with recent findings by Liu and coworkers, who showed that siRNA knock-down of GPC3 expression in ovarian cancer cells induces increases in cell proliferation as well as in migration and invasion properties [40]. Several studies indicate that aberrant Hh signaling plays a role in thyroid neoplasia [41,42], and *GPC3* expression has also been investigated in thyroid cancer [43]. In sharp contrast to our findings, immunohistochemistry studies by Yamanaka and coworkers indicate that *GPC3 is* overexpressed in PTC. We do not have an explanation for this discrepancy. However, it is important to note that our immunohistochemical data are consistent with the mRNA levels we observed in a different cohort of samples. Moreover, data in the Human Protein Atlas (http://www.proteinatlas.org/) also show that GPC3 protein expression is reduced in PTC compared with NT, and, in a recent study of TCGA, *GPC3* mRNA levels were markedly reduced in PTC compared with NT [44]. Collectively, these data indicate that GPC3 might exert an oncosuppressor function that is lost during thyroid tumorigenesis. Our findings suggest that assessment of *GPC3* expression could be used to discriminate between PTC and NT.

In conclusion, our study provides evidence of significant dysregulation of several putative PAX8 target genes in PTC, supporting the notion that PAX8-regulated molecular cascades play important roles in thyroid tumorigenesis. The molecular mechanisms underlying this dysregulation are still unclear and should be addressed in future studies.

## Supporting Information

S1 Fig Correlation between *PAX8* and thyroid-specific gene mRNA levels in the 36 PTCs. R and p values have been calculated by the Spearman rank correlation test.
(TIF)

S2 Fig *LCN2* mRNA levels in TCGA dataset. (A) *LCN2* mRNA levels in distinct ATA risk groups. (B) ROC curve of *LCN2* mRNA levels as predictor of ATA high risk group.
(TIF)

S1 Table Expression of putative *PAX8* target genes in the 36 PTCs stratified by histotypes. (DOC)

S2 Table Correlation between mRNA levels of *PAX8* and its putative target genes in the 36 PTCs. (DOC)

S3 Table Expression of putative *PAX8* target genes in PTCs stratified by *BRAF* mutational status (TCGA dataset). (DOC)

S4 Table Expression of *PAX8* target genes in PTCs stratified by ATA risk (TCGA dataset). (DOC)

## Abbreviations

NT: normal thyroid
PTC: papillary thyroid cancer
