# Supplementary figures and images for: Expression of PAX8 Target Genes in Papillary Thyroid Carcinoma
Source: PLoS One. 2016 Jun 1;11(6):e0156658. doi: 10.1371/journal.pone.0156658 (PMC4889154; doi:10.1371/journal.pone.0156658)

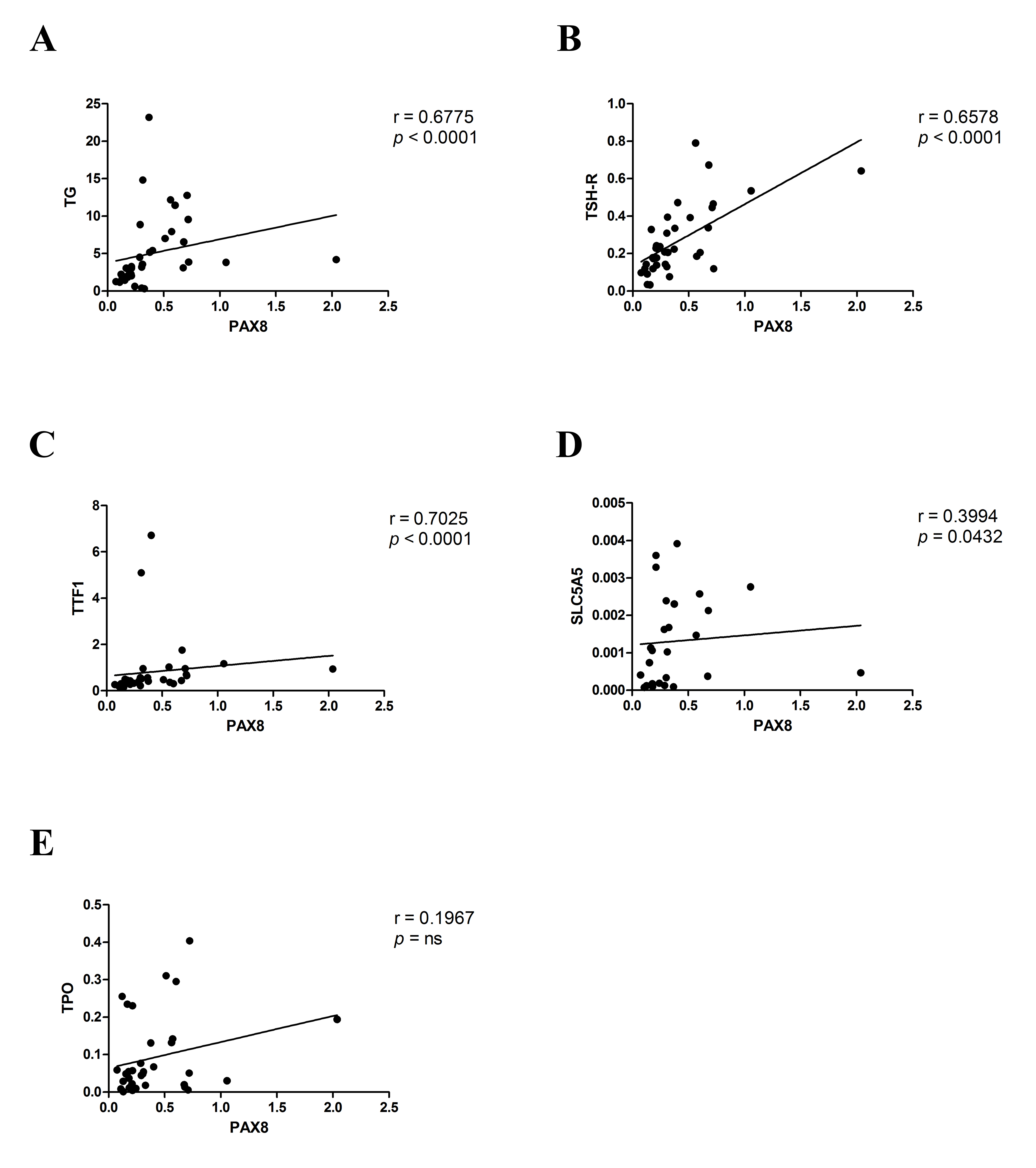

Supplement: S1 Fig — R and p values have been calculated by the Spearman rank correlation test. (TIF) [file pone.0156658.s001.tif]

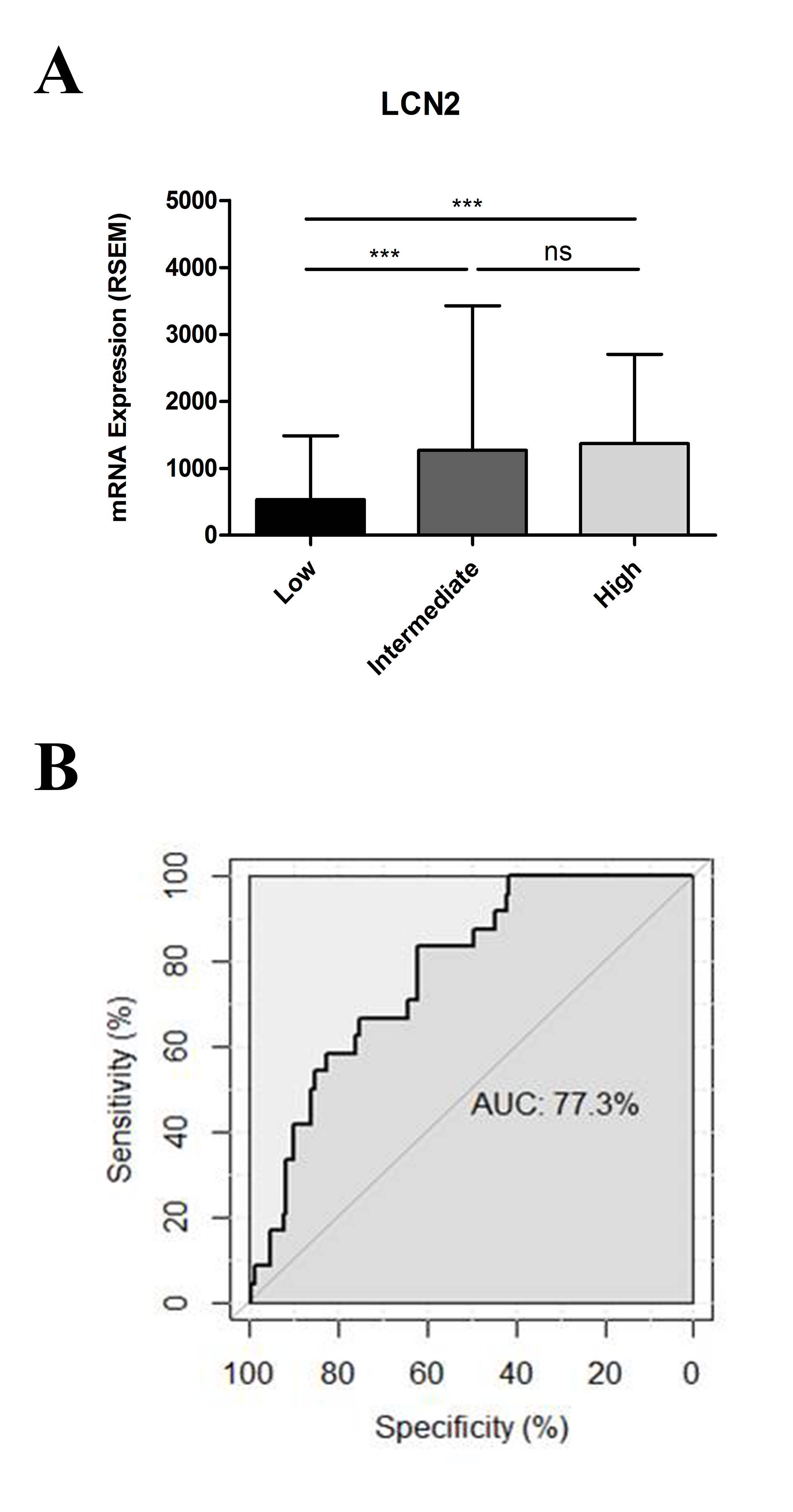

Supplement: S2 Fig — (A) LCN2 mRNA levels in distinct ATA risk groups. (B) ROC curve of LCN2 mRNA levels as predictor of ATA high risk group. (TIF) [file pone.0156658.s002.tif]
