# Supplementary material for: Expression of PAX8 Target Genes in Papillary Thyroid Carcinoma
Source: PLoS One. 2016 Jun 1;11(6):e0156658. doi: 10.1371/journal.pone.0156658 (PMC4889154; doi:10.1371/journal.pone.0156658)
Supplement: S1 Table — (DOC) [file pone.0156658.s003.doc]

**Supplemental Table 1. Expression of putative *PAX8* target genes in the 36 PTCs stratified by histotypes.**

|  |  |  |  | ***p-value*** | | | |
| --- | --- | --- | --- | --- | --- | --- | --- |
| **Gene** | **NT (n=18)** | **PTC-cl**  **(n=31)** | **PTC-fv (n=5)** | ***Kruskal-Wallis*** | **CT-PTC**  ***vs* Normal Tissues** | **FV-PTC**  ***vs* Normal Tissues** | **CT-PTC**  ***vs***  **FV-PTC** |
| **Upregulated in Pax8 knock-out mice*** | | | | | | | |
| ***CA3*** | 1±0.462 | 1.323±0.760 | 0.972±0.304 | ns | ns | ns | ns |
| ***FSTL1*** | 1±0.496 | 1.178±0.876 | 0.386±0.233 | 0.0204 | ns | <0.05 | <0.05 |
| ***GPC3*** | 1±0.626 | 0.072±0.096 | 0.037±0.046 | <0.0001 | <0.001 | <0.001 | ns |
| ***LCN2*** | 1±0.857 | 49.850±81.840 | 2.161±2.894 | <0.0001 | <0.001 | ns | <0.05 |
| ***LGALS1*** | 1±0.643 | 3.200±3.017 | 0.512±0.268 | <0.0001 | <0.001 | ns | <0.001 |
| ***LUM*** | 1±0.743 | 2.728±9.321 | 1.773±3.948 | ns | ns | ns | ns |
| ***SCD1*** | 1±0.948 | 3.470±2.693 | 0.735±0.382 | <0.0001 | <0.001 | ns | <0.05 |
| **Downregulated in Pax8 knock-out mice*** | | | | | | | |
| ***ATP1B1*** | 1±0.493 | 0.724±0.353 | 0.694±0.144 | ns | ns | ns | ns |
| ***KCNIP3*** | 1±0.829 | 0.276±0.220 | 0.845±0.690 | <0.0001 | <0.001 | ns | ns |
| ***NFKBIA*** | 1±0.429 | 1.106±1.334 | 1.067±0.848 | ns | ns | ns | ns |
| ***PRLR*** | 1±0.820 | 0.418±0.308 | 0.390±0.323 | 0.0069 | <0.01 | ns | ns |

mRNA levels are expressed as mean ± SD. PTC, Papillary Thyroid Carcinoma; FV-PTC, follicular variant; CT-PTC, classical-type.

p values were obtained by Kruskal–Wallis test with post-hoc Dunn’s multiple comparison test.

* Data from Marotta et al., 2014.
