# Supplementary material for: Expression of PAX8 Target Genes in Papillary Thyroid Carcinoma
Source: PLoS One. 2016 Jun 1;11(6):e0156658. doi: 10.1371/journal.pone.0156658 (PMC4889154; doi:10.1371/journal.pone.0156658)
Supplement: S2 Table — (DOC) [file pone.0156658.s004.doc]

**Supplemental Table 2. Correlation between mRNA levels of *PAX8* and its putative target genes in the 36 PTCs.**

| **Gene** | **r^a^** | **p-value** |
| --- | --- | --- |
| ***ATP1B1*** | 0.5012 | 0.0018 |
| ***CAR3*** | 0.5013 | 0.0022 |
| ***FSTL1*** | 0.4337 | 0.0082 |
| ***GPC3*** | 0.2269 | 0.1899 |
| ***KCNIP3*** | 0.7109 | <0.0001 |
| ***LCN2*** | 0.02423 | 0.8901 |
| ***LGALS1*** | 0.3622 | 0.0383 |
| ***LUM*** | -0.2621 | 0.1226 |
| ***NFKBIA*** | 0.6244 | <0.0001 |
| ***PRLR*** | 0.3543 | 0.0971 |
| ***SCD1*** | -0.02703 | 0.8757 |

^a^ Spearman rho rank correlation coefficient**.**
