# Supplementary material for: Expression of PAX8 Target Genes in Papillary Thyroid Carcinoma
Source: PLoS One. 2016 Jun 1;11(6):e0156658. doi: 10.1371/journal.pone.0156658 (PMC4889154; doi:10.1371/journal.pone.0156658)
Supplement: S3 Table — (DOC) [file pone.0156658.s005.doc]

**Supplemental Table 3. Expression of putative *PAX8* target genes in PTCs stratified by *BRAF* mutational status (TCGA dataset).**

| **Gene** | ***BRAF-wt***  **(n=258)** | ***BRAF*^V600E^**  **(n=228)** | **Fold**  **Change** | **p-value** | **adj**  **p-value** |
| --- | --- | --- | --- | --- | --- |
| **Upregulated genes in Pax8 knock-out mice*** | | | | | |
| ***CA3*** | 77.4±50.9 | 92.0±211.0 | 1.2 | ns | ns |
| ***FSTL1*** | 2749.1±1542.4 | 3473.0±2114.2 | 1.3 | <0.0001 | 0.0011 |
| ***GPC3*** | 36.2±68.5 | 25.5±41.9 | 0.7 | 0.011 | ns |
| ***LCN2*** | 575.3±1276.6 | 1555.5±2205.5 | 2.7 | <0.0001 | 0.0011 |
| ***LGALS1*** | 3550.7±4251.4 | 4459.8±3125.2 | 1.3 | <0.0001 | 0.0011 |
| ***LUM*** | 1398.0±2630.3 | 2977.3±4568.2 | 2.1 | <0.0001 | 0.0011 |
| ***SCD1*** | 479.5±603.5 | 814.8±674.3 | 1.7 | <0.0001 | 0.0011 |
| **Downregulated genes in Pax8 knock-out mice*** | | | | | |
| ***ATP1B1*** | 13194.4±6031.2 | 13458.6±4193.5 | 1.0 | 0.066 | ns |
| ***KCNIP3*** | 1196.5±1089.8 | 407.9±225.9 | 0.3 | <0.0001 | 0.0011 |
| ***NFKBIA*** | 2156.7±1021.2 | 1852.7±872.5 | 0.9 | 0.0029 | 0.0319 |
| ***PRLR*** | 21.8±60.8 | 16.0±53.3 | 0.7 | ns | ns |

Expression levels are reported as RSEM mean value ± SD.

p-values were obtained by Mann-Whitney test.

adjusted p-values were evaluated using the Bonferroni method.

wt, wild type; ns, not significant.

* Data from Marotta et al., 2014.
