# Supplementary material for: Expression of PAX8 Target Genes in Papillary Thyroid Carcinoma
Source: PLoS One. 2016 Jun 1;11(6):e0156658. doi: 10.1371/journal.pone.0156658 (PMC4889154; doi:10.1371/journal.pone.0156658)
Supplement: S4 Table — (DOC) [file pone.0156658.s006.doc]

**Supplemental Table 4. Expression of *PAX8* target genes in PTCs stratified by ATA risk (TCGA dataset).**

| **Gene** | ***Low Risk***  ***(n=166)*** | ***Intermediate Risk***  ***(n=252)*** | ***High Risk (n=24)*** | ***Fold Change*** | ***p-value*** | |
| --- | --- | --- | --- | --- | --- | --- |
|  |  |  |  |  | ***Kruskal-Wallis*** | ***Dunn's test*** |
| **Upregulated genes in Pax8 knock-out mice*** | | | | | | |
| ***CA3*** | 72.2±41.0 | 89.2±198.7 | 87.4±62.6 | 1.2 ^a^ | ns | ns ^a^ |
|  |  |  |  | 1.2 ^b^ |  | ns ^b^ |
|  |  |  |  | 1.0 ^c^ |  | ns ^c^ |
| ***FSTL1*** | 2689.6±1269.4 | 3231.5±1902.2 | 4058.8±3998.7 | 1.2 ^a^ | 0.0036 | <0.01 ^a^ |
|  |  |  |  | 1.5 ^b^ |  | ns ^b^ |
|  |  |  |  | 1.3 ^c^ |  | ns ^c^ |
| ***GPC3*** | 33.6±54.5 | 28.5±48.9 | 16.7±23.8 | 0.8 ^a^ | ns | ns ^a^ |
|  |  |  |  | 0.5 ^b^ |  | ns ^b^ |
|  |  |  |  | 0.6 ^c^ |  | ns ^c^ |
| ***LCN2*** | 532.4±953.3 | 1269.3±2159.3 | 1367.2±1333.6 | 2.4 ^a^ | <0.0001 | <0.001 ^a^ |
|  |  |  |  | 2.6 ^b^ |  | <0.001 ^b^ |
|  |  |  |  | 1.1 ^c^ |  | ns ^c^ |
| ***LGALS1*** | 2925.2±2972.0 | 4482.3±4202.2 | 4760.7±3537.0 | 1.5 ^a^ | <0.0001 | <0.001 ^a^ |
|  |  |  |  | 1.6 ^b^ |  | <0.05 ^b^ |
|  |  |  |  | 1.1 ^c^ |  | ns ^c^ |
| ***LUM*** | 1001.4±1565.2 | 2588.4±4021.1 | 4487.4±8204.6 | 2.6 ^a^ | <0.0001 | <0.001 ^a^ |
|  |  |  |  | 4.5 ^b^ |  | <0.05 ^b^ |
|  |  |  |  | 1.7 ^c^ |  | ns ^c^ |
| ***SCD1*** | 470.1±456.0 | 732.0±655.0 | 737.0±614.4 | 1.6 ^a^ | <0.0001 | <0.001 ^a^ |
|  |  |  |  | 1.6 ^b^ |  | <0.05 ^b^ |
|  |  |  |  | 1.0 ^c^ |  | ns ^c^ |
| **Downregulated genes in Pax8 knock-out mice*** | | | | | | |
| ***ATP1B1*** | 13188.8±5797.7 | 13499.1±4817.9 | 12075.7±4769.4 | 1.0^a^ | ns | ns ^a^ |
|  |  |  |  | 0.9^b^ |  | ns ^b^ |
|  |  |  |  | 0.9^c^ |  | ns ^c^ |
| ***KCNIP3*** | 1125.8±1094.2 | 650.8±673.0 | 585.5±439.7 | 0.6 ^a^ | <0.0001 | <0.001 ^a^ |
|  |  |  |  | 0.5 ^b^ |  | <0.01 ^b^ |
|  |  |  |  | 0.9 ^c^ |  | ns ^c^ |
| ***NFKBIA*** | 2021.6±889.9 | 1967.2±967.5 | 1922.9±935.7 | 1.0 ^a^ | ns | ns ^a^ |
|  |  |  |  | 1.0 ^b^ |  | ns ^b^ |
|  |  |  |  | 1.0 ^c^ |  | ns ^c^ |
| ***PRLR*** | 19.0±49.6 | 15.9±48.2 | 20.6±63.9 | 0.8 ^a^ | ns | ns ^a^ |
|  |  |  |  | 1.1 ^b^ |  | ns ^b^ |
|  |  |  |  | 1.3 ^c^ |  | ns ^c^ |

Expression levels are reported as RSEM mean value ± SD.

p-values were obtained by Kruskal–Wallis test with post hoc Dunn’s multiple comparison test.

^a^ Intermediate Risk PTCs vs. Low Risk PTCs.

^b^ High Risk PTCs vs. Low Risk PTCs.

^c^ Intermediate Risk PTCs vs. High Risk PTCs.

ns, not significant.

* Data from Marotta et al., 2014.
